# A Real-World Prospective Study of the Safety and Effectiveness of the Loop Open Source Automated Insulin Delivery System

**DOI:** 10.1089/dia.2020.0535

**Published:** 2021-04-20

**Authors:** John W. Lum, Ryan J. Bailey, Victoria Barnes-Lomen, Diana Naranjo, Korey K. Hood, Rayhan A. Lal, Brandon Arbiter, Adam S. Brown, Daniel J. DeSalvo, Jeremy Pettus, Peter Calhoun, Roy W. Beck

**Affiliations:** ^1^ Jaeb Center for Health Research, Tampa, Florida, USA.; ^2^ Department of Pediatrics and Medicine, Stanford University School of Medicine, Stanford, California, USA.; ^3^ Tidepool, Palo Alto, California, USA.; ^4^ Section of Pediatric Diabetes and Endocrinology, Baylor College of Medicine, Houston, Texas, USA.; ^5^ Division of Endocrinology, University of California, San Diego, San Diego, California, USA.

**Keywords:** Type 1 diabetes, Automated insulin delivery, Closed-loop control, Continuous glucose monitors, Safety

## Abstract

***Objective:*** To evaluate the safety and effectiveness of the Loop Do-It-Yourself automated insulin delivery system.

***Research Design and Methods:*** A prospective real-world observational study was conducted, which included 558 adults and children (age range 1–71 years, mean HbA1c 6.8% ± 1.0%) who initiated Loop either on their own or with community-developed resources and provided data for 6 months.

***Results:*** Mean time-in-range 70–180 mg/dL (TIR) increased from 67% ± 16% at baseline (before starting Loop) to 73% ± 13% during the 6 months (mean change from baseline 6.6%, 95% confidence interval [CI] 5.9%–7.4%; *P* < 0.001). TIR increased in both adults and children, across the full range of baseline HbA1c, and in participants with both high- and moderate-income levels. Median time <54 mg/dL was 0.40% at baseline and changed by −0.05% (95% CI −0.09% to −0.03%, *P* < 0.001). Mean HbA1c was 6.8% ± 1.0% at baseline and decreased to 6.5% ± 0.8% after 6 months (mean difference = −0.33%, 95% CI −0.40% to −0.26%, *P* < 0.001). The incidence rate of reported severe hypoglycemia events was 18.7 per 100 person-years, a reduction from the incidence rate of 181 per 100 person-years during the 3 months before the study. Among the 481 users providing Loop data at 6 months, median continuous glucose monitoring use was 96% (interquartile range [IQR] 91%–98%) and median time Loop modulating basal insulin was at least 83% (IQR 73%–88%).

***Conclusions:*** The Loop open source system can be initiated with community-developed resources and used safely and effectively by adults and children with type 1 diabetes.

## Introduction

Before closed-loop systems becoming commercially available, “Do-It-Yourself” closed-loop systems were developed by individuals with a personal interest in automating insulin delivery for type 1 diabetes (T1D). One such system called “Loop” was developed by Nate Racklyeft, Pete Schwamb, and others in the open-source diabetes software community in 2015.

Loop is an open-source iOS app that runs on an iPhone. The software is a hybrid closed-loop controller utilizing model prediction that anticipates future glucose based on the effects of delivered insulin, user-entered carbohydrates, and two forms of short-term adaptation dubbed “glucose momentum” and “retrospective correction.” The effects ofcarbohydrates are based on user-specified insulin-to-carbohydrate ratio and insulin sensitivity factor (ISF), whereas the insulin effect is determined by the ISF. Loop then alters insulin delivery to attempt to drive the blood sugar toward a user-specified glucose target. In the most common implementation, Loop alters insulin delivery by instructing the insulin pump to temporarily increase or decrease basal insulin delivery.

Originally, Loop supported versions of Medtronic insulin pumps that could receive unsecure remote commands. The Insulet Omnipod pump became another option in April 2019 after months of internal testing by the development community. A RileyLink serves as a bridge between the iPhone's Bluetooth and the sub-gigahertz radio frequency used by these pumps. The system is compatible with Dexcom and Medtronic continuous glucose monitors (CGMs). An Apple Watch optionally may be used with the system for additional user interaction. Additionally, users can enter meal absorption times, adjust glucose targets and insulin needs during exercise, and track their insulin delivery from the Loop app or through logging software such as Nightscout or Tidepool.

Loop is being used worldwide by up to 9000 individuals based on the RileyLink order history (personal communication, Jeremy Lucas, founder of GetRileyLink.org, February 2020), despite limited data on the system's safety and efficacy. To provide this crucial information, we conducted an observational, longitudinal study of existing and new Loop users. Herein, we report on the data provided by new Loop users from the time they initiated Loop for up to 6 months.

## Methods

The study was conducted in a real-world setting, outside of the clinic, with all data provided directly by study participants. The protocol was approved by the Institutional Review Board of the JAEB Center for Health Research. The protocol is available at https://public.jaeb.org/datasets and summarized on clinicaltrials.gov (NCT03838900).

The study included adults and children with T1D who were U.S. residents. The existence of the study was publicized on websites and at the time of ordering a RileyLink (required for Loop operation). Interested individuals were directed to the study website for information about the study where electronic informed consent was obtained from participants ≥18 years of age and the legally authorized representative for participants <18 years of age who provided assent. The analysis herein included only participants who were initiating Loop to understand Loop's impact from a baseline without prior use of Loop. Enrollment was open between January 2019 and August 2019. The data collection ended in April 2020 such that all participants had the opportunity to complete 6 months of follow-up, which comprises the dataset reported herein.

Participants provided demographic and socioeconomic information, information about their medical history and medications, diabetes history and management, and height and weight. Weekly, participants received a text and/or email prompt to report any device issues or serious adverse events, including diabetic ketoacidosis (DKA), severe hypoglycemia, and hospitalizations. All DKA and severe hypoglycemia events reported after enrollment were reviewed. Confirmation of DKA required hospitalization for at least one night. Confirmation of severe hypoglycemia required a description that was consistent with the participant being impaired cognitively to the point that he/she was unable to treat himself/herself; being unable to verbalize his/her needs; being incoherent, disoriented, and/or combative; or having experienced seizure or loss of consciousness. At baseline, participants were asked to report the number of such episodes that had occurred in the prior 3 months.

A fingerstick blood sample was obtained for HbA1c measurement, using a collection kit mailed directly to the study participant, at baseline and after 3, 6, and 12 months and mailed to a central laboratory (University of Minnesota Advanced Research and Diagnostic Laboratory). Of the 1412 samples sent to the laboratory, 215 (15%) were determined to not be analyzed when received by the laboratory, typically due to suspected temperature-related loss of sample integrity or because too much time had passed since sample collection. Results from 5 (0.4%) of the 1197 analyzed samples were considered unreliable and excluded. Samples were considered unreliable if the HbA1c was less than 5.0% (31 mmol/mol) and the difference with the glucose management indicator (GMI) estimate of HbA1c^1^ was inconsistent with HbA1c-GMI differences at other time points, or the HbA1c-GMI difference was >2% and not consistent with differences at other time points. The quality of life/psychosocial and treatment satisfaction surveys were completed at baseline and after 3, 6, and 12 months. Focus group sessions, which will be described in a separate report, were held within the first 3 months of initiating Loop and at the end of the study; additionally, interviews were held with 20 participants who discontinued Loop.

At study entry, prior pump and CGM data were obtained when available. During the study, Loop system data were written to Apple Health and then continuously streamed to Tidepool, where the data were aggregated. There was no standardization of how the Loop system was to be used. Participants were asked to export Loop's Issue Report monthly, which provided data on pump settings, Loop version, and Loop settings, including therapy settings.

### Statistical methods

The study sample was a convenience sample and was not based on statistical principles. To be included in the cohort for analysis, participants had to (1) not have started Loop, or used it for <7 days at the time of enrollment; (2) provided at least 50 records of Loop basal insulin data (represents 4–8 h of Loop use) or at least 1 Loop device issue report after starting Loop; and (3) provided at least 336 h (14 days) of CGM data in the first 182 days after starting Loop.

All eligible participants were included in the safety analysis. CGM data provided before the date of Loop initiation (minimum of 168 h) were used to calculate metrics at baseline, whereas all data provided from the date of Loop initiation to 182 days after Loop initiation were used to calculate outcomes at 6 months of follow-up. For normally distributed outcomes, a paired *t*-test was used to evaluate differences between baseline and 6 months follow-up. If outcomes were skewed, a Wilcoxon signed rank test was used instead. For binary outcomes, McNemar's test was used to evaluate differences between baseline and follow-up.

The prespecified primary efficacy outcomes were percent time in range 70–180 mg/dL, time >180 mg/dL, mean glucose, time <70 mg/dL, time <54 mg/dL, and HbA1c. For comparisons of these outcomes between baseline and follow-up, the family-wise type 1 error rate was controlled at a two-sided alpha of 0.05 using a hierarchical approach. Secondary efficacy outcomes tested were the glucose standard deviation, glucose coefficient of variation, time in range 70–140 mg/dL, time >250 mg/dL, high blood glucose index, low blood glucose index, area under the curve above 180 mg/dL (area under the receiver operating characteristic curve >180 mg/dL), area over the curve below 70 mg/dL (area over the curve [AOC] <70 mg/dL), the rate of hypoglycemia events below 54 mg/dL per week, and the percentage of participants meeting international glucose consensus targets to T1D (nonpregnant).^2^ For comparisons of secondary outcomes, the false discovery rate was controlled using the Benjamini–Hochberg procedure with <0.05 as a threshold for statistical significance.

Additional analyses assessed change from baseline in the first 3 months and second 3 months after starting Loop. Analysis of CGM outcomes were conducted separately for daytime (6:00 AM–11:59 PM) and nighttime (12:00 AM–5:59 AM).

All *P*-values and confidence intervals (CIs) reported are two-sided. All analyses were conducted using SAS software, version 9.4 (SAS Institute, Inc.).

## Results

Of the 799 new Loop users who were enrolled and provided electronic consent, 241 did not meet the inclusion criteria (Supplementary Fig. S1). Most (93%) of the exclusions were due to no data or insufficient data being provided. The age range of the 558 eligible participants was 1–71 years. Baseline HbA1c averaged 6.8% ± 1.0% (51 ± 10.9 mmol/mol) (including 84 participants with HbA1c ≥7.5% [58 mmol/mol]). Thirty-six (6%) used only a Medtronic pump during the study, whereas 502 (90%) used only the Omnipod pump and 20 (4%) used both at some point during the study follow-up (Table 1). At least 168 h (1 week) of baseline CGM data (before starting Loop) were available for 447 (80%) of the 558 participants. There were several variants of Loop utilized in this study (Supplementary Table S1 footnote).

**Table 1.** Participant Characteristics

	Overall	<7 years	7–<14 years	14–<25 years	25–<50 years	≥50 years
	n = 558	n = 67	n = 169	n = 87	n = 192	n = 43
Age at enrollment (years), *n*	558	67	169	87	192	43
Mean ± SD	23 ± 16	5 ± 1	10 ± 2	18 ± 3	35 ± 7	57 ± 5
Range	1–71	1–6	7–13	14–24	25–49	50–71
Female	310/547 (57%)	31/65 (48%)	80/163 (49%)	51/86 (59%)	122/190 (64%)	26/43 (60%)
Race						
White	484/531 (91%)	51/64 (80%)	144/156 (92%)	79/84 (94%)	168/184 (91%)	42/43 (98%)
Black/African American	5/531 (<1%)	2/64 (3%)	0/156 (0%)	2/84 (2%)	1/184 (<1%)	0/43 (0%)
Hispanic or Latino	21/531 (4%)	4/64 (6%)	7/156 (4%)	2/84 (2%)	7/184 (4%)	1/43 (2%)
Asian	9/531 (2%)	2/64 (3%)	3/156 (2%)	1/84 (1%)	3/184 (2%)	0/43 (0%)
More than one race	12/531 (2%)	5/64 (8%)	2/156 (1%)	0/84 (0%)	5/184 (3%)	0/43 (0%)
BMI (kg/m^2^), *n*^a^	266	—	—	34	190	42
Mean ± SD	26 ± 5	—	—	26 ± 5	27 ± 5	26 ± 4
BMI percentile, *n*^b^	268	60	157	51	—	—
Median (quartiles)	75% (51%, 89%)	84% (69%, 96%)	74% (50%, 86%)	61% (32%, 82%)	—	—
Age at diabetes diagnosis (years), *n*	543	63	162	85	190	43
Mean ± SD	11 ± 10	3 ± 1	6 ± 3	10 ± 5	16 ± 10	22 ± 13
Duration of T1D (years), *n*	543	63	162	85	190	43
Median (quartiles)	8 (4, 20)	2 (2, 4)	5 (3, 7)	7 (4, 12)	20 (12, 26)	37 (26, 45)
Highest level of education^c^						
Less than high school	5/544 (<1%)	1/65 (2%)	3/164 (2%)	1/84 (1%)	0/188 (0%)	0/43 (0%)
High school graduate/some college	71/544 (13%)	2/65 (3%)	15/164 (9%)	21/84 (25%)	26/188 (14%)	7/43 (16%)
Bachelor's degree	220/544 (40%)	15/65 (23%)	65/164 (40%)	34/84 (40%)	93/188 (49%)	13/43 (30%)
Master's degree	164/544 (30%)	33/65 (51%)	50/164 (30%)	18/84 (21%)	50/188 (27%)	13/43 (30%)
Professional/doctorate degree	84/544 (15%)	14/65 (22%)	31/164 (19%)	10/84 (12%)	19/188 (10%)	10/43 (23%)
Annual household income						
<$25,000	6/498 (1%)	0/59 (0%)	0/156 (0%)	1/73 (1%)	4/177 (2%)	1/33 (3%)
$25,000–<50,000	27/498 (5%)	0/59 (0%)	2/156 (1%)	9/73 (12%)	11/177 (6%)	5/33 (15%)
$50,000–<75,000	40/498 (8%)	4/59 (7%)	9/156 (6%)	4/73 (5%)	18/177 (10%)	5/33 (15%)
$75,000–<100,000	77/498 (15%)	15/59 (25%)	16/156 (10%)	8/73 (11%)	34/177 (19%)	4/33 (12%)
$100,000 or more	348/498 (70%)	40/59 (68%)	129/156 (83%)	51/73 (70%)	110/177 (62%)	18/33 (55%)
Type of insurance						
Private health insurance	513/546 (94%)	59/65 (91%)	158/164 (96%)	81/84 (96%)	180/190 (95%)	35/43 (81%)
Public health insurance	31/546 (6%)	6/65 (9%)	5/164 (3%)	3/84 (4%)	9/190 (5%)	8/43 (19%)
Other	2/546 (<1%)	0/65 (0%)	1/164 (<1%)	0/84 (0%)	1/190 (<1%)	0/43 (0%)
Pump used with Loop						
Medtronic Minimed	36/558 (6%)	2/67 (3%)	1/169 (<1%)	2/87 (2%)	23/192 (12%)	8/43 (19%)
Insulet Omnipod	502/558 (90%)	64/67 (96%)	166/169 (98%)	82/87 (94%)	157/192 (82%)	33/43 (77%)
Both	20/558 (4%)	1/67 (1%)	2/169 (1%)	3/87 (3%)	12/192 (6%)	2/43 (5%)
Severe hypo events in the previous 3 months (baseline, before Loop)						
Total events	247	53	92	24	43	35
Experienced at least 1 event	97/545 (18%)	16/65 (25%)	32/163 (20%)	12/85 (14%)	24/189 (13%)	13/43 (30%)
Events per participant						
0	448 (82%)	49 (75%)	131 (80%)	73 (86%)	165 (87%)	30 (70%)
1	51 (9%)	5 (8%)	19 (12%)	7 (8%)	14 (7%)	6 (14%)
2	17 (3%)	2 (3%)	3 (2%)	2 (2%)	7 (4%)	3 (7%)
≥3	29 (5%)	9 (14%)	10 (6%)	3 (4%)	3 (2%)	4 (9%)
DKA events in the previous 3 months (baseline, before Loop)						
Total events	23	1	8	3	8	3
Experienced at least 1 event	13/537 (2%)	1/63 (2%)	4/158 (3%)	3/85 (4%)	3/189 (2%)	2/42 (5%)
Events per participant						
0	524 (98%)	62 (98%)	154 (97%)	82 (96%)	186 (98%)	40 (95%)
1	8 (1%)	1 (2%)	2 (1%)	3 (4%)	1 (<1%)	1 (2%)
2	1 (<1%)	0 (0%)	0 (0%)	0 (0%)	0 (0%)	1 (2%)
≥3	4 (<1%)	0 (0%)	2 (1%)	0 (0%)	2 (1%)	0 (0%)
Follows a low-carb diet	85/545 (16%)	7/64 (11%)	7/163 (4%)	5/86 (6%)	49/189 (26%)	17/43 (40%)
Previously used automated insulin delivery system	53/547 (10%)	1/65 (2%)	9/164 (5%)	11/86 (13%)	21/189 (11%)	11/43 (26%)
Systems used in the past						
Medtronic 670G	37/53 (70%)	0/1 (0%)	5/9 (56%)	7/11 (64%)	18/21 (86%)	7/11 (64%)
OpenAPS	8/53 (15%)	0/1 (0%)	2/9 (22%)	2/11 (18%)	2/21 (10%)	2/11 (18%)
AndroidAPS	1/53 (2%)	0/1 (0%)	1/9 (11%)	0/11 (0%)	0/21 (0%)	0/11 (0%)
Other	10/53 (19%)	1/1 (100%)	3/9 (33%)	2/11 (18%)	2/21 (10%)	2/11 (18%)
Type of insulin currently using						
Apidra (glulisine)	8/526 (2%)	1/63 (2%)	3/158 (2%)	1/82 (1%)	2/180 (1%)	1/43 (2%)
Fiasp (rapid aspart)	46/526 (9%)	5/63 (8%)	8/158 (5%)	9/82 (11%)	21/180 (12%)	3/43 (7%)
Humalog (lispro)	255/526 (48%)	32/63 (51%)	79/158 (50%)	35/82 (43%)	83/180 (46%)	26/43 (60%)
Novolog (aspart)	216/526 (41%)	25/63 (40%)	67/158 (42%)	37/82 (45%)	74/180 (41%)	13/43 (30%)
Regular insulin	1/526 (<1%)	0/63 (0%)	1/158 (<1%)	0/82 (0%)	0/180 (0%)	0/43 (0%)
Bolus calculation method						
Bolus calculator	234/547 (43%)	25/65 (38%)	67/164 (41%)	37/86 (43%)	84/189 (44%)	21/43 (49%)
Count carbohydrates	226/547 (41%)	31/65 (48%)	83/164 (51%)	34/86 (40%)	69/189 (37%)	9/43 (21%)
Experience	68/547 (12%)	4/65 (6%)	7/164 (4%)	12/86 (14%)	32/189 (17%)	13/43 (30%)
Other	19/547 (3%)	5/65 (8%)	7/164 (4%)	3/86 (3%)	4/189 (2%)	0/43 (0%)
GLP1-analog inhibitor use	19/555 (3%)	0/67 (0%)	1/168 (<1%)	2/87 (2%)	14/190 (7%)	2/43 (5%)
SGLT2-inhibitor use	13/555 (2%)	0/67 (0%)	0/168 (0%)	1/87 (1%)	12/190 (6%)	0/43 (0%)
Baseline HbA1c, *n*	378	43	110	54	135	36
Mean ± SD, % (mmol/mol)	6.8 ± 1.0 (51 ± 10.9)	6.8 ± 0.8 (51 ± 8.7)	7.0 ± 0.8 (53 ± 8.7)	7.1 ± 1.3 (54 ± 14.2)	6.7 ± 1.0 (50 ± 10.9)	6.6 ± 0.8 (49 ± 8.7)

^a^ Reported for subjects 18 years of age or older.

^b^ Reported for subjects younger than 18 years of age.

^c^ For subjects younger than 18, indicates highest parental education.

APS, artificial pancreas system; BMI, body mass index; DKA, diabetic ketoacidosis; GLP-1, glucagon-like peptide 1; SD, standard deviation; SGLT2, sodium-glucose cotransporter-2; T1D, type 1 diabetes.

### Efficacy outcomes

**FIG. 1.**
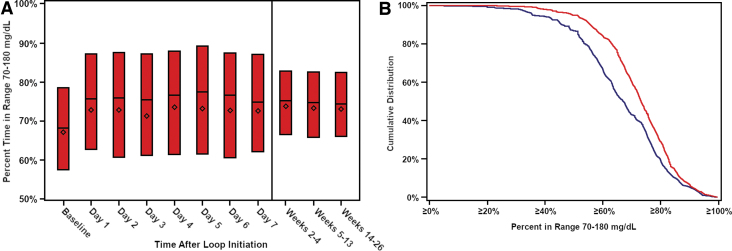
**(A)** Time in range 70–180 mg/dL in the first 4 weeks after Loop initiation. **(B)** Cumulative distribution of time in range at baseline and follow-up. Solid blue line represents baseline. Solid red line represents months 1–6.

**FIG. 2.**
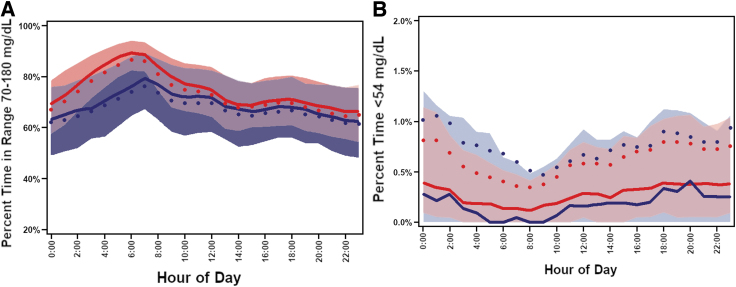
**(A)** Time in range 70–180 mg/dL over a 24 h period. Solid blue line represents median baseline. Solid blue dot represents mean baseline. Shaded blue represents baseline quartiles. Solid red line represents median months 1–6. Solid red dot represents mean months 1–6. Shaded red represents months 1–6 quartiles. **(B)** Time <54 mg/dL over a 24 h period. Solid blue line represents median baseline. Solid blue dot represents mean baseline. Shaded blue represents baseline quartiles. Solid red line represents median months 1–6. Solid red dot represents mean months 1–6. Shaded red represents months 1–6 quartiles.

Mean time-in-range (TIR) increased from 67% ± 16% at baseline (CGM data before starting Loop) to 73% ± 13% during the 6 months (mean change from baseline 6.6%, 95% CI 5.9%–7.4%; *P* < 0.001; the percentage of participants with TIR >70% increased from 44% to 63% (*P* < 0.001) (Table 2). The treatment effect was evident on the first day of Loop use and remained consistent over the 6 months of follow-up (Fig. 1A). The cumulative distribution of TIR at baseline and during follow-up is shown in Figure 1B. Over the 24 h of the day, the effect on TIR and hyperglycemia was more evident overnight and early morning although present throughout the day (Fig. 2A and Supplementary Fig. S2). Daytime (6 AM–12 midnight) mean TIR was 67% ± 16% at baseline versus 72% ± 13% during follow-up, and nighttime (12 midnight–6 AM) was 66% ± 17% versus 76% ± 14%, respectively (Supplementary Table S2). Time <54 mg/dL was low at baseline (median 0.40%) but nevertheless was significantly reduced using Loop (mean change −0.05%, 95% CI −0.09% to −0.03%, *P* < 0.001) during follow-up (Fig. 2B). Significant reductions from baseline were seen in all hyperglycemia metrics and most hypoglycemia metrics (Table 2). Over the 6 months, 274 (49%) participants had both TIR >70% plus time <54 mg/dL <1% compared with 144 (32%) at baseline (*P* < 0.001). Results during the first 3 months and second 3 months on Loop were similar to the overall 6 months (Supplementary Table S3).

**Table 2.** Glycemic Outcomes at Baseline and Over 6 Months Follow-Up

	Baseline (*n* = 447)	Over 6 months follow-up (*n* = 558)	Change from baseline to follow-up difference (95% CI)^a^	P^a^
Hours of CGM data, mean ± SD	1852 ± 923 (77 ± 38 days)	3815 ± 816 (159 ± 34 days)	—	—
Primary outcomes^b^				
Percent time in range 70–180 mg/dL, mean ± SD	67% ± 16%	73% ± 13%	6.6% (5.9% to 7.4%)	<0.001
Percent time >180 mg/dL, mean ± SD	29% ± 17%	24% ± 14%	−6.2% (−7.0% to −5.4%)	<0.001
Mean glucose (mg/dL), mean ± SD	155 ± 29	147 ± 23	−10 (−11 to −8)	<0.001
Percent time <70 mg/dL, median (IQR)	2.9% (1.3%, 5.2%)	2.8% (1.3%, 4.7%)	−0.2% (−0.4% to −0.1%)	0.002
Percent time <54 mg/dL, median (IQR)	0.40% (0.13%, 0.96%)	0.36% (0.15%, 0.85%)	−0.05% (−0.09% to −0.03%)	<0.001
HbA1c mean ± SD, % (mmol/mol)^c^	6.8 ± 1.0	6.5 ± 0.8	−0.33% (−0.40% to −0.26%)	<0.001
	51 ± 10.9	48 ± 8.7	[−3.6 (−4.4 to −2.80)]	
Secondary continuous outcomes,^d^ median (IQR)				
Percent time in range 70–140 mg/dL	44% (35%, 57%)	52% (42%, 61%)	6.7% (6.0% to 7.5%)	<0.001
Percent time >250 mg/dL	6% (2%, 13%)	5% (2%, 9%)	−1.8% (−2.2% to −1.5%)	<0.001
High blood glucose index	6.1 (3.6, 9.1)	4.9 (3.2, 7.1)	−1.2 (−1.4 to −1.0)	<0.001
AUC >180 mg/dL	13 (6, 23)	10 (5, 17)	−3.3 (−4.0 to −2.7)	<0.001
Low blood glucose index	0.9 (0.5, 1.4)	0.9 (0.5, 1.4)	0.01 (−0.03 to 0.04)	0.67
AOC <70 mg/dL	0.3 (0.1, 0.5)	0.2 (0.1, 0.5)	−0.03 (−0.04 to −0.01)	<0.001
Rate of hypoglycemia events per week (<54 mg/dL)^d,e^	0.8 (0.2, 2.0)	0.7 (0.3, 1.7)	−0.16 (−0.19 to −0.13)	<0.001
Glucose coefficient of variation (%), median (IQR)	37% (33%, 41%)	37% (33%, 40%)	−0.3% (−0.6% to −0.0%)	0.05
Glucose standard deviation (mg/dL), median (IQR)	57 (48, 68)	54 (45, 62)	−3.5 (−4.1 to −2.9)	<0.001
Secondary binary outcomes,^d^*n* (%)				
Time in range 70–180 mg/dL >70%	198 (44%)	353 (63%)	21% (17% to 25%)	<0.001
Time below 70 mg/dL <4%	275 (62%)	379 (68%)	6% (2% to 9%)	0.001
Time below 54 mg/dL <1%	341 (76%)	452 (81%)	5% (2% to 9%)	0.002
Time in range 70–180 mg/dL >70% plus time below 70 mg/dL <4%	97 (22%)	209 (37%)	17% (13% to 21%)	<0.001
Time in range 70–180 mg/dL >70% plus time below 54 mg/dL <1%	144 (32%)	274 (49%)	19% (15% to 23%)	<0.001
HbA1c < 7.0% (<53 mmol/mol)^c^	220 (58%)	332 (75%)	19% (14% to 23%)	<0.001
HbA1c improvement from baseline ≥0.5% (≥5.5 mmol/mol)^c^	—	178 (40%)	—	—

^a^ For continuous outcomes, *P*-values estimated from paired *t*-test or Wilcoxon signed-rank test using complete-case analysis (*n* = 447 for CGM analysis and *n* = 335 for HbA1c analysis) and CIs for the mean or median difference are reported. For the rate of hypoglycemia events per week, *P*-value estimated from a Poisson regression model and CI for difference in event rates is reported. For binary outcomes, *P*-values estimated from McNemar's test and Wald CIs for the difference in proportions are reported.

^b^ Hierarchical approach used to control for the family-wise error rate.

^c^ For HbA1c outcomes *n* = 378 at baseline and *n* = 443 at follow-up.

^d^ *P*-values and CIs adjusted using the adaptive Benjamini–Hochberg procedure to control the false discovery rate.

^e^ A hypoglycemic event was defined as at least 15 continuous minutes with CGM readings <54 mg/dL. The end of an event was defined as at least 15 continuous minutes with CGM readings ≥70 mg/dL.

AOC, area over the curve; AUC, area under the receiver operating characteristic curve; CGM, continuous glucose monitoring; CI, confidence interval; IQR, interquartile range.

The beneficial effect on CGM metrics was seen throughout the age range of participants (Supplementary Table S4). As seen in Supplementary Table S1, improvement in TIR was seen across the range of baseline HbA1c, with greater amount of improvement occurring in those with higher baseline HbA1c (and lower baseline TIR) and higher TIR levels achieved in those with higher baseline TIR. Medtronic pump users were older and more likely to have prior automated insulin delivery (AID) use compared with Omnipod pump users (Supplementary Table S5), but glycemic results were similar by pump manufacturer (Supplementary Table S6).

Mean HbA1c was 6.8% ± 1.0% (51 ± 10.9 mmol/mol) at baseline and decreased to 6.5% ± 0.8% (48 ± 8.7 mmol/mol) after 6 months (mean difference = −0.33%, 95% CI −0.40% to −0.26% [−3.6, 95% CI −4.4 to −2.8 mmol/mol], *P* < 0.001). The percentages of participants with HbA1c level <7.0% (<53 mmol/mol) were 58% at baseline and 75% at 6 months (*P* < 0.001, Table 2).

### Glucose monitoring and closed-loop system use

Seventy-seven (14%) participants stopped providing Loop data during the first 6 months, of whom 15 (3%) provided information indicating that they had discontinued Loop. Reasons for discontinuing are indicated in Supplementary Table S7. For the other 62, it is not known whether they stopped using Loop or just stopped providing data. The most common reported issues with use of Loop were problems with connectivity and communication (27% of total issues reported) and hardware damage/failure (10% of issues reported) (Supplementary Table S8).

Among the 481 participants who provided Loop data through 6 months, median CGM use during 6 months was 96% (interquartile range [IQR] 91%–98%) and median time that Loop was modulating the basal rate was at least 83% (IQR 73%–88%) (Supplementary Table S9). The 83% number represents a lower bound for percent time in closed-loop with device data not permitting differentiation between closed-loop, open-loop, or other system status 17% of the time. Among all subjects included, the mean total daily insulin over 6 months was 0.70 ± 0.40 U/kg, with modulated basal insulin representing 54% of insulin delivered (Supplementary Table S10).

### Safety outcomes

Supplementary Table S11 shows the safety outcomes according to age groups. For the 14,755 weekly surveys that could possibly be completed, median percent completion per participant was 89% (IQR 67%–93%). There were no cases of confirmed DKA. During the 6 months of the study, 35 (6%) participants experienced a total of 51 confirmed severe hypoglycemia events (incidence rate 18.7 per 100 person-years), with 28 (5%) participants experiencing the event in the first 3 months (incidence rate 27.3 per 100 person-years). Five of the 51 events involved seizure or loss of consciousness (incidence rate 1.8 per 100 person-years). One of the 51 events was attributed to the use of Loop, however, this event was not associated with a seizure or loss of consciousness. For comparison, 97 (18%) participants reported at least one severe hypoglycemia event in the 3 months before entering the study (incidence rate 181 per 100 person-years). The frequency of severe hypoglycemia events during the study was substantially higher in participants who had experienced an event in the 3 months before the study (Supplementary Table S12).

## Discussion

Open-source AID systems are used by many adults and children to automate insulin delivery for management of T1D, with at least 9000 having used Loop. The study data reflect real-world use of Loop in that there was no guidance provided by the study as to how Loop was to be used and no formal customer support for troubleshooting. The study participants initiated Loop with community-developed resources. TIR, which on average was already at a high level before starting Loop, increased further; and time <54 mg/dL decreased. Improvement in TIR occurred immediately after starting Loop and was sustained on average over 6 months. The benefits of Loop were seen in both adults and children, across the full range of baseline HbA1c, and with both high- and moderate-income levels.

The improvement in TIR during this observational study was similar in magnitude to that reported in randomized controlled trials of other closed-loop systems after accounting for differences among study cohorts in baseline HbA1c levels.^3–6^ For HbA1c levels above 7.0% (53 mmol/mol) with baseline TIR of about 60%, the improvement in TIR observed with different systems has been remarkably consistent, about 10% overall, with greater improvement seen overnight than during the day. For higher baseline TIR, the magnitude of improvement tends to be less and for lower baseline TIR, it tends to be more.

The incidence rate of severe hypoglycemia of 18.7 events per 100 person-years is higher than what has been reported in the afore-referenced studies of other closed-loop systems, some (but not all) of which excluded individuals with recent severe hypoglycemia. There are several possible explanations: (1) this could reflect our frequent ascertainment of severe hypoglycemia through weekly text prompts; (2) differences in study design: the Loop study was real-world and virtual compared with other studies that had structured protocols with close clinical oversight of closed-loop system use by study staff; or (3) higher prestudy risk of this cohort for severe hypoglycemia, possibly due to a more hyperglycemia-avoidant approach to diabetes management. Indeed, prior severe hypoglycemia has been shown to be the strongest predictor of future severe hypoglycemia, similar to the finding in this study.^7^ The study data suggest that the use of Loop substantially reduced the risk of severe hypoglycemia in this cohort, since the rate of severe hypoglycemia was much lower during the first 3 months of Loop use than what was reported for the 3 months before starting Loop (27.3 vs. 181.3 per 100-person years); however, this must be interpreted in the context of different data collection methods used to capture the prestudy and on-study reports of severe hypoglycemia events.

The strengths of the study include the large sample size, the real-world approach to the protocol, the prospective data collection, the inclusion in the cohort of individuals who were new Loop users, the availability of pre-Loop CGM data for most participants to establish a baseline for comparison with glycemic metrics while using Loop, and the wide age range of study participants from infants to older adults. The main limitations are the lack of concurrent control group, and self-selection bias in participants starting on Loop. Indeed, most of the cohort had HbA1c levels <7.0% (<53 mmol/mol) before starting Loop and most were of high socioeconomic status. This limits the generalizability of the results. Despite the skewness of these factors relative to the population of individuals with T1D, the sample size was sufficiently large that the number of participants with high HbA1c (40 with baseline HbA1c ≥ 8.0% [≥64 mmol/mol]), and moderate family income (33 < $50,000) was not inconsequential. In these subgroups, the benefit of Loop appeared comparable to those with lower HbA1c and higher income. We have uncertainty as to the percentage of participants who started Loop and discontinued use of Loop before 6 months, since for 62 participants we are unable to determine if Loop was discontinued or if the participant just stopped providing data. The percentage could be as low as 3% or as high as 14%. We also have uncertainty about the percentage of time that Loop was in closed-loop mode during the 6 months, automatically modulating the basal rate. The percentage is no lower than 83% and is almost certainly higher, but we could not differentiate between possible system states in the remaining 17%, including the user actively turning off closed-loop, communication errors between components or other component failures causing reversion to open-loop, or the system delivering an unaltered open-loop scheduled basal rate as designed (e.g., when glucose is within the user set “correction range”). We believe that the completeness of the dataset is quite robust for a real-world observational study, but the amount of missing data, nevertheless, reflects a limitation of this type of study.

In summary, this real-world study has demonstrated that the Loop open-source hybrid closed-loop system can be safely self-initiated and used by adults and children with T1D and reduced time in range without increasing hypoglycemia. Tidepool is developing a commercial version of Loop (“Tidepool Loop”), which will rely on the data generated in this study to support FDA clearance.

## Supplementary Material

Supplemental data

Supplemental data

Supplemental data

Supplemental data

Supplemental data

Supplemental data

Supplemental data

Supplemental data

Supplemental data

Supplemental data

Supplemental data

Supplemental data

Supplemental data

Supplemental data
