# Supplementary material for: A Real-World Prospective Study of the Safety and Effectiveness of the Loop Open Source Automated Insulin Delivery System
Source: Diabetes Technol Ther. 2021 Apr 20;23(5):367–75. doi: 10.1089/dia.2020.0535 (PMC8080906; doi:10.1089/dia.2020.0535)
Supplement: Supplemental data [file Supp_Fig1.docx]

# Supplemental Figure S1. Study Flowchart

Enrolled

N=799

Ineligible

N=241

- Never started Loop or did not provide sufficient Loop data (N=177)
- Used Spike app prior to the study (N=14)
- Live outside U.S. (N=2)
- Did not provide at least 336 hours of CGM data during follow-up (N=48)

Analyzed

N=558

Stopped Using Loop or Stopped Providing Loop Data*

N=35

Actively Using Loop at End of 3 Months (91 days) Follow-Up

N=523

Actively Using Loop at End of 6 Months (182 days) Follow-Up

N=481

Stopped Using Loop or Stopped Providing Loop Data**

N=42

*8 participants indicated that they discontinued using Loop

**7 participants indicated that they discontinued using Loop
