# Supplementary material for: A Real-World Prospective Study of the Safety and Effectiveness of the Loop Open Source Automated Insulin Delivery System
Source: Diabetes Technol Ther. 2021 Apr 20;23(5):367–75. doi: 10.1089/dia.2020.0535 (PMC8080906; doi:10.1089/dia.2020.0535)
Supplement: Supplemental data [file Supp_Table1.docx]

# Supplemental Table S1. Time in Range 70-180 mg/dL by Sub-Groups ^a^

|  | N | Baseline | N | Over 6 Months | P-Value ^b^ |
| --- | --- | --- | --- | --- | --- |
| **Diabetes Duration (yrs)** |  |  |  |  |  |
| <5 | 156 | 69% (59%, 78%) | 196 | 73% (66%, 80%) | <0.001 |
| ≥5 | 281 | 68% (57%, 79%) | 347 | 76% (66%, 83%) | <0.001 |
| **Gender** |  |  |  |  |  |
| Female | 253 | 68% (56%, 78%) | 310 | 75% (66%, 82%) | <0.001 |
| Male | 186 | 69% (59%, 79%) | 236 | 74% (66%, 82%) | <0.001 |
| **Annual Household Income** |  |  |  |  |  |
| <$25,000 | 5 | 76% (59%, 78%) | 6 | 79% (74%, 82%) | 0.12 |
| $25,000 - <$50,000 | 20 | 63% (55%, 74%) | 27 | 72% (64%, 79%) | <0.001 |
| $50,000 - <$75,000 | 32 | 73% (60%, 83%) | 40 | 79% (69%, 84%) | <0.001 |
| $75,000 - <$100,000 | 69 | 70% (57%, 78%) | 77 | 74% (68%, 84%) | <0.001 |
| ≥$100,000 | 274 | 67% (58%, 78%) | 348 | 74% (66%, 82%) | <0.001 |
| **BMI ^c^** |  |  |  |  |  |
| Not Overweight/Obese | 255 | 69% (58%, 78%) | 313 | 74% (66%, 82%) | <0.001 |
| Overweight | 101 | 69% (59%, 81%) | 129 | 77% (67%, 84%) | <0.001 |
| Obese | 71 | 65% (53%, 77%) | 92 | 74% (66%, 79%) | <0.001 |
| **Pump Type** |  |  |  |  |  |
| Medtronic Only | 25 | 71% (61%, 76%) | 35 | 78% (72%, 84%) | <0.001 |
| Omnipod Only | 404 | 68% (57%, 79%) | 496 | 74% (66%, 82%) | <0.001 |
| Both | 15 | 70% (62%, 79%) | 20 | 80% (75%, 83%) | 0.01 |
| **CGM Used** |  |  |  |  |  |
| Dexcom G4/G5 | 56 | 68% (57%, 78%) | 70 | 74% (65%, 83%) | <0.001 |
| Dexcom G6 | 384 | 68% (58%, 78%) | 477 | 74% (66%, 82%) | <0.001 |
| **Apple Watch User** |  |  |  |  |  |
| No | 219 | 67% (57%, 78%) | 286 | 73% (66%, 81%) | <0.001 |
| Yes | 222 | 69% (58%, 79%) | 262 | 76% (67%, 84%) | <0.001 |
| **Baseline Time in Range** |  |  |  |  |  |
| <70% | 249 | 59% (52%, 64%) | 249 | 68% (61%, 73%) | <0.001 |
| >70% | 198 | 79% (76%, 85%) | 198 | 83% (79%, 88%) | <0.001 |
| **Baseline HbA1c (%)** |  |  |  |  |  |
| <6.5 | 127 | 79% (75%, 85%) | 148 | 83% (77%, 88%) | <0.001 |
| 6.5-6.9 | 64 | 69% (64%, 76%) | 75 | 75% (71%, 80%) | <0.001 |
| 7.0-7.4 | 64 | 61% (57%, 68%) | 75 | 71% (66%, 75%) | <0.001 |
| 7.5-7.9 | 35 | 56% (51%, 64%) | 44 | 67% (59%, 72%) | <0.001 |
| ≥8.0 | 31 | 42% (33%, 49%) | 40 | 55% (48%, 63%) | <0.001 |
| **Loop Branch** ^d^ |  |  |  |  |  |
| Katie | 65 | 69% (59%, 79%) | 86 | 75% (68%, 83%) | <0.001 |
| Main-Line | 334 | 68% (57%, 78%) | 402 | 75% (66%, 82%) | <0.001 |

^a^ Values are Median (Q1, Q3)

^b^ P-values obtained from paired t-test.

^c^ Not obese/overweight defined as a BMI of <25 for adults (age ≥18) and a percentile of <85% for children (age <18). Overweight defined as a BMI of 25-<30 for adults and a BMI percentile of 85%-95% for children. Obese defined as a BMI >30 for adults and a BMI percentile >95% for children.

^d^ Loop Branch refers to variations that exist in the open-source code. “Main-Line” includes participants using Loop versions 1.9.3, 1.9.4, 1.9.5, 1.9.6, 1.10.0, 1.10.1, 1.10.3, 2.0, and 2.1.0
