# Supplementary figures and images for: A Real-World Prospective Study of the Safety and Effectiveness of the Loop Open Source Automated Insulin Delivery System
Source: Diabetes Technol Ther. 2021 Apr 20;23(5):367–75. doi: 10.1089/dia.2020.0535 (PMC8080906; doi:10.1089/dia.2020.0535)

# Supplemental Figure S2. Time > 180 mg/dL by Hour over 24 Hours


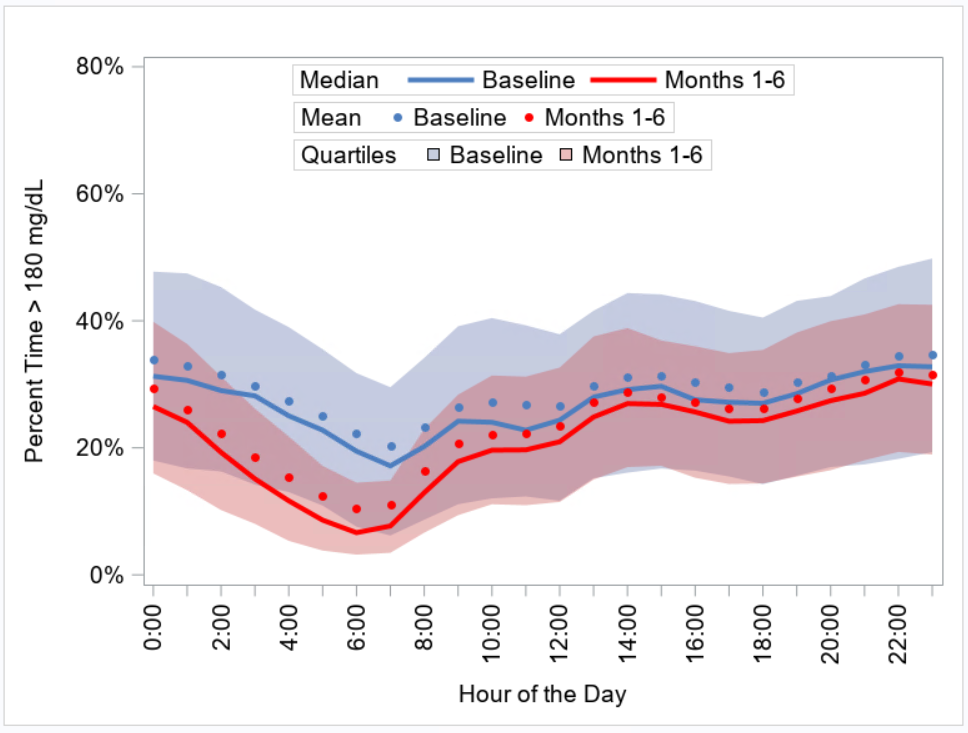

Supplement: Supplemental data [file Supp_Fig2.docx]
