# Supplementary material for: A Real-World Prospective Study of the Safety and Effectiveness of the Loop Open Source Automated Insulin Delivery System
Source: Diabetes Technol Ther. 2021 Apr 20;23(5):367–75. doi: 10.1089/dia.2020.0535 (PMC8080906; doi:10.1089/dia.2020.0535)
Supplement: Supplemental data [file Supp_Table2.docx]

# Supplemental Table S2. CGM Metrics by Time of Day

|  | Baseline  N=447 | Over 6 Months  N=558 | P-Value ^a^ |
| --- | --- | --- | --- |
| **Daytime ^b^** |  |  |  |
| Time in Range 70-180 mg/dL *mean ± SD* | 67% ± 16% | 72% ± 13% | <0.001 |
| Time >180 mg/dL *mean ± SD* | 29% ± 17% | 24% ± 14% | <0.001 |
| Mean Glucose *mean ± SD* | 155 ± 30 | 148 ± 24 | <0.001 |
| Time <70 mg/dL *median (IQR)* | 2.9% (1.2%, 5.1%) | 2.9% (1.4%, 4.7%) | 0.01 |
| Time <54 mg/dL *median (IQR)* | 0.38% (0.10%, 0.84%) | 0.35% (0.13%, 0.77%) | 0.001 |
| Percent Time >250 mg/dL *median (IQR)* | 6% (2%, 13%) | 5% (2%, 10%) | <0.001 |
| High Blood Glucose Index *median (IQR)* | 6.2 (3.5, 9.1) | 5.2 (3.3, 7.5) | <0.001 |
| AUC >180 mg/dL *median (IQR)* | 13 (6, 23) | 11 (5, 18) | <0.001 |
| Low Blood Glucose Index *median (IQR)* | 0.9 (0.5, 1.4) | 0.9 (0.5, 1.4) | 0.74 |
| AOC <70 mg/dL *median (IQR)* | 0.23 (0.10, 0.50) | 0.24 (0.11, 0.43) | 0.001 |
| Hypoglycemia Events per Week (<54 mg/dL) *median (IQR) ^c^* | 0.7 (0.1, 1.9) | 0.7 (0.2, 1.7) | <0.001 |
| Glucose Coefficient of Variation (%) *median (IQR)* | 37% (33%, 40%) | 37% (33%, 41%) | 0.48 |
| Glucose Standard Deviation (mg/dL) *median (IQR)* | 57 (47, 68) | 55 (46, 63) | <0.001 |
| **Nighttime ^d^** |  |  |  |
| Time in Range 70-180 mg/dL *mean ± SD* | 66% ± 17% | 76% ± 14% | <0.001 |
| Time >180 mg/dL *mean ± SD* | 30% ± 19% | 21% ± 14% | <0.001 |
| Mean Glucose *mean ± SD* | 156 ± 32 | 142 ± 24 | <0.001 |
| Time <70 mg/dL *median (IQR)* | 2.6% (1.0%, 5.5%) | 2.4% (1.2%, 4.6%) | 0.007 |
| Time <54 mg/dL *median (IQR)* | 0.39% (0.07%, 1.0%) | 0.33% (0.11%, 0.84%) | <0.001 |
| Percent Time >250 mg/dL *median (IQR)* | 6% (2%, 13%) | 3% (1%, 7%) | <0.001 |
| High Blood Glucose Index *median (IQR)* | 6.1 (3.6, 9.5) | 4.0 (2.4, 6.2) | <0.001 |
| AUC >180 mg/dL *median (IQR)* | 12 (5, 25) | 7 (3, 14) | <0.001 |
| Low Blood Glucose Index *median (IQR)* | 0.8 (0.4, 1.5) | 0.9 (0.5, 1.4) | 0.26 |
| AOC <70 mg/dL *median (IQR)* | 0.23 (0.07, 0.54) | 0.22 (0.09, 0.43) | 0.002 |
| Hypoglycemia Events per Week (<54 mg/dL) *median (IQR) ^c^* | 0.8 (0.0, 2.0) | 0.6 (0.2, 1.6) | <0.001 |
| Glucose Coefficient of Variation (%) *median (IQR)* | 36% (32%, 41%) | 35% (31%, 38%) | <0.001 |
| Glucose Standard Deviation (mg/dL) *median (IQR)* | 56 (45, 68) | 49 (40, 58) | <0.001 |

^a^ P-values are calculated using paired t-tests or Wilcoxon sign-rank tests comparing baseline to 6 month outcomes. P-values are adjusted for multiple comparisons using the adaptive Benjamini-Hochberg procedure

^b^ Daytime between 6:00 AM and 11:59 PM

^c^ A hypoglycemic event was defined as at least 15 continuous minutes with CGM readings <54 mg/dL. The end of an event was defined as at least 15 continuous minutes with CGM readings ≥70 mg/dL

^d^ Nighttime between 12:00 AM and 5:59 AM
