# Supplementary material for: A Real-World Prospective Study of the Safety and Effectiveness of the Loop Open Source Automated Insulin Delivery System
Source: Diabetes Technol Ther. 2021 Apr 20;23(5):367–75. doi: 10.1089/dia.2020.0535 (PMC8080906; doi:10.1089/dia.2020.0535)
Supplement: Supplemental data [file Supp_Table3.docx]

# Supplemental Table S3. Glycemic Outcomes at 3 and 6 Months of Follow-Up

|  | Baseline  N=447 | 1-3 Months ^a^  N=553 | P-Value ^b^ | 4-6 Months ^a^  N=527 | P-Value ^b^ |
| --- | --- | --- | --- | --- | --- |
| % Time in Range – Mean ± SD | 67% ± 16% | 73% ± 13% | <0.001 | 73% ± 13% | <0.001 |
| % Time >180 mg/dL – Mean ± SD | 29% ± 17% | 23% ± 14% | <0.001 | 23% ± 14% | <0.001 |
| Mean Glucose (mg/dL) – Mean ± SD | 155 ± 29 | 146 ± 23 | <0.001 | 146 ± 24 | <0.001 |
| % Time <70 mg/dL – Median (Quartiles) | 2.9% (1.3%, 5.2%) | 2.7% (1.4%, 4.7%) | 0.002 | 2.6% (1.3%, 4.7%) | 0.003 |
| % Time <54 mg/dL – Median (Quartiles) | 0.40% (0.13%, 0.96%) | 0.35% (0.14%, 0.81%) | <0.001 | 0.33% (0.14%, 0.82%) | <0.001 |
| HbA1c (%) – Mean ± SD ^c^ | 6.8 ± 1.0 | 6.5 ± 0.8 | <0.001 | 6.5 ± 0.8 | <0.001 |
| % Time >250 mg/dL – Median (Quartiles) | 6% (2%, 13%) | 5% (2%, 9%) | <0.001 | 5% (2%, 9%) | <0.001 |
| HBGI – Median (Quartiles) | 6.1 (3.6, 9.1) | 4.8 (3.0, 7.1) | <0.001 | 4.8 (3.0, 7.1) | <0.001 |
| AUC >180 mg/dL – Median (Quartiles) | 13 (6, 23) | 10 (5, 17) | <0.001 | 10 (5, 17) | <0.001 |
| LBGI – Median (Quartiles) | 0.9 (0.5, 1.4) | 0.9 (0.5, 1.4) | 0.74 | 0.9 (0.5, 1.4) | 0.72 |
| AOC <70 mg/dL – Median (Quartiles) | 0.26 (0.10, 0.52) | 0.23 (0.10, 0.44) | <0.001 | 0.23 (0.11, 0.44) | <0.001 |
| Weekly Hypo Event Rate – Median (Quartiles) ^d^ | 0.8 (0.2, 2.0) | 0.7 (0.2, 1.7) | <0.001 | 0.6 (0.2, 1.6) | <0.001 |
| Glucose SD (mg/dL) – Median (Quartiles) | 57 (48, 68) | 53 (45, 62) | <0.001 | 53 (45, 62) | <0.001 |
| Glucose CV (%) – Median (Quartiles) | 37% (33%, 41%) | 36% (33%, 40%) | <0.001 | 37% (33%, 40%) | 0.02 |

^a^ Participants must have provided at least 336 hours of data in the respective follow-up period to be included in the analysis

^b^ P-values are calculated using paired t-tests or Wilcoxon sign-rank tests comparing baseline to 3- and 6-month outcomes.

^c^ For HbA1c, N=378 at baseline, N=371 at month 3, N=443 at month 6.

^d^ A hypoglycemic event was defined as at least 15 continuous minutes with CGM readings <54 mg/dL. The end of an event was defined as at least 15 continuous minutes with CGM readings ≥70 mg/dL
