# Supplementary material for: A Real-World Prospective Study of the Safety and Effectiveness of the Loop Open Source Automated Insulin Delivery System
Source: Diabetes Technol Ther. 2021 Apr 20;23(5):367–75. doi: 10.1089/dia.2020.0535 (PMC8080906; doi:10.1089/dia.2020.0535)
Supplement: Supplemental data [file Supp_Table4.docx]

# Supplemental Table S4. Glycemic Outcomes by Age Group ^a^

|  | N | Baseline | N | Over 6 Months | P-Value ^b^ |
| --- | --- | --- | --- | --- | --- |
| **% Time in Range 70-180 mg/dL** |  |  |  |  |  |
| Age <7 Years | 51 | 67% (58%, 78%) | 67 | 73% (65%, 80%) | <0.001 |
| Age 7-<14 Years | 135 | 64% (55%, 75%) | 169 | 69% (64%, 77%) | <0.001 |
| Age 14-<25 Years | 65 | 63% (57%, 73%) | 87 | 71% (63%, 76%) | <0.001 |
| Age 25-<50 Years | 157 | 72% (61%, 82%) | 192 | 80% (72%, 86%) | <0.001 |
| Age ≥50 Years | 39 | 78% (62%, 84%) | 43 | 79% (70%, 88%) | <0.001 |
| **% Time >180 mg/dL** |  |  |  |  |  |
| Age <7 Years | 51 | 28% (16%, 40%) | 67 | 21% (14%, 32%) | <0.001 |
| Age 7-<14 Years | 135 | 33% (21%, 41%) | 169 | 26% (18%, 33%) | <0.001 |
| Age 14-<25 Years | 65 | 33% (23%, 42%) | 87 | 28% (21%, 36%) | <0.001 |
| Age 25-<50 Years | 157 | 23% (12%, 36%) | 192 | 16% (10%, 23%) | <0.001 |
| Age ≥50 Years | 39 | 17% (10%, 36%) | 43 | 16% (10%, 28%) | 0.01 |
| **Mean Glucose (mg/dL)** |  |  |  |  |  |
| Age <7 Years | 51 | 149 (135, 171) | 67 | 142 (134, 159) | <0.001 |
| Age 7-<14 Years | 135 | 159 (142, 174) | 169 | 150 (135, 161) | <0.001 |
| Age 14-<25 Years | 65 | 161 (145, 174) | 87 | 154 (142, 166) | <0.001 |
| Age 25-<50 Years | 157 | 146 (129, 165) | 192 | 137 (124, 148) | <0.001 |
| Age ≥50 Years | 39 | 139 (125, 164) | 43 | 134 (125, 155) | 0.02 |
| **% Time <70 mg/dL** |  |  |  |  |  |
| Age <7 Years | 51 | 3.1% (1.6%, 5.2%) | 67 | 3.2% (1.8%, 5.4%) | 0.06 |
| Age 7-<14 Years | 135 | 2.9% (1.3%, 5.1%) | 169 | 3.0% (1.8%, 5.2%) | 0.72 |
| Age 14-<25 Years | 65 | 2.2% (1.2%, 4.4%) | 87 | 2.0% (1.0%, 3.3%) | 0.02 |
| Age 25-<50 Years | 157 | 3.1% (1.1%, 5.7%) | 192 | 2.7% (1.2%, 4.9%) | 0.007 |
| Age ≥50 Years | 39 | 3.7% (1.3%, 5.6%) | 43 | 2.7% (1.2%, 4.2%) | <0.001 |
| **% Time <54 mg/dL** |  |  |  |  |  |
| Age <7 Years | 51 | 0.48% (0.17%, 0.81%) | 67 | 0.41% (0.18%, 0.88%) | 0.29 |
| Age 7-<14 Years | 135 | 0.40% (0.16%, 0.94%) | 169 | 0.42% (0.19%, 0.89%) | 0.23 |
| Age 14-<25 Years | 65 | 0.36% (0.15%, 0.76%) | 87 | 0.28% (0.13%, 0.57%) | 0.003 |
| Age 25-<50 Years | 157 | 0.40% (0.09%, 1.05%) | 192 | 0.35% (0.12%, 0.90%) | <0.001 |
| Age ≥50 Years | 39 | 0.41% (0.09%, 1.10%) | 43 | 0.32% (0.11%, 0.80%) | 0.23 |
| **HbA1c (%)** |  |  |  |  |  |
| Age <7 Years | 43 | 6.5 (6.4, 7.3) | 43 | 6.5 (6.0, 6.8) | 0.005 |
| Age 7-<14 Years | 110 | 7.0 (6.3, 7.5) | 138 | 6.6 (6.2, 7.2) | <0.001 |
| Age 14-<25 Years | 54 | 6.9 (6.4, 7.5) | 67 | 6.8 (6.2, 7.2) | 0.02 |
| Age 25-<50 Years | 135 | 6.5 (6.0, 7.2) | 153 | 6.1 (5.7, 6.6) | <0.001 |
| Age ≥50 Years | 36 | 6.5 (6.1, 7.2) | 41 | 6.6 (5.8, 6.9) | 0.004 |
| **% CGM Use ^c^** |  |  |  |  |  |
| Age <7 Years | - | - | 55 | 97% (94%, 99%) | - |
| Age 7-<14 Years | - | - | 148 | 95% (87%, 97%) | - |
| Age 14-<25 Years | - | - | 73 | 95% (88%, 97%) | - |
| Age 25-<50 Years | - | - | 166 | 97% (92%, 98%) | - |
| Age ≥50 Years | - | - | 39 | 96% (85%, 98%) | - |
| **% Time Loop Modulated Basal ^c^** |  |  |  |  |  |
| Age <7 Years | - | - | 55 | 85% (75%, 89%) | - |
| Age 7-<14 Years | - | - | 148 | 82% (73%, 87%) | - |
| Age 14-<25 Years | - | - | 73 | 80% (68%, 87%) | - |
| Age 25-<50 Years | - | - | 166 | 84% (75%, 89%) | - |
| Age ≥50 Years | - | - | 39 | 83% (69%, 89%) | - |

^a^ Values are median (Q1, Q3)

^b^ P-values estimated from paired t-test

^c^ Only includes participants who were providing Loop data at the end of 6 months follow-up
