# Supplementary material for: A Real-World Prospective Study of the Safety and Effectiveness of the Loop Open Source Automated Insulin Delivery System
Source: Diabetes Technol Ther. 2021 Apr 20;23(5):367–75. doi: 10.1089/dia.2020.0535 (PMC8080906; doi:10.1089/dia.2020.0535)
Supplement: Supplemental data [file Supp_Table5.docx]

# Supplemental Table S5. Participant Characteristics by Pump

|  | Medtronic Only  N=36 | Omnipod Only  N=502 |
| --- | --- | --- |
| Age (Years) – N | 36 | 502 |
| mean ± SD | 36 ± 15 | 22 ± 16 |
| Female-N (%) | 26/36 (72%) | 270/491 (55%) |
| Diabetes Duration (Years) – N | 35 | 488 |
| mean ± SD | 22 ± 11 | 12 ± 12 |
| Annual Income |  |  |
| <25K | 1/28 (4%) | 4/451 (<1%) |
| 25K-<50K | 1/28 (4%) | 23/451 (5%) |
| 50K-<75K | 4/28 (14%) | 35/451 (8%) |
| 75K-<100K | 2/28 (7%) | 73/451 (16%) |
| ≥100K | 20/28 (71%) | 316/451 (70%) |
| Severe Hypo Event in 3 Months Prior to Enrollment | 9/36 (25%) | 86/489 (18%) |
| DKA Event in 3 Months Prior to Enrollment | 0/35 (0%) | 12/482 (2%) |
| Prior AID Use | 12/36 (33%) | 33/491 (7%) |
