# Supplementary material for: A Real-World Prospective Study of the Safety and Effectiveness of the Loop Open Source Automated Insulin Delivery System
Source: Diabetes Technol Ther. 2021 Apr 20;23(5):367–75. doi: 10.1089/dia.2020.0535 (PMC8080906; doi:10.1089/dia.2020.0535)
Supplement: Supplemental data [file Supp_Table6.docx]

# Supplemental Table S6. Glycemic Outcomes by Pump Used During Study ^a^

|  | Medtronic Only | | Omnipod Only | |
| --- | --- | --- | --- | --- |
|  | Baseline  N=25 | Over 6 Months  N=35 | Baseline  N=404 | Over 6 Months  N=496 |
| % Time in Range 70-180 mg/dL | 71% (61%, 76%) | 78% (72%, 84%) | 68% (57%, 79%) | 74% (66%, 82%) |
| % Time >180 mg/dL | 23% (19%, 36%) | 19% (10%, 24%) | 28% (16%, 40%) | 22% (14%, 32%) |
| Mean Glucose (mg/dL) | 146 (141, 164) | 140 (124, 150) | 152 (135, 171) | 143 (131, 159) |
| % Time <70 mg/dL | 3.3% (1.3%, 4.3%) | 2.7% (1.6%, 5.0%) | 2.9% (1.3%, 5.2%) | 2.8% (1.3%, 4.7%) |
| % Time <54 mg/dL | 0.52% (0.16%, 0.91%) | 0.41% (0.19%, 0.92%) | 0.40% (0.13%, 0.95%) | 0.35% (0.15%, 0.82%) |
| HbA1c (%) ^b^ | 6.6 (5.7, 7.2) | 6.4 (6.0, 6.7) | 6.7 (6.2, 7.3) | 6.5 (6.0, 7.0) |
| % CGM Use ^c^ | - | 94% (87%, 97%) | - | 96% (91%, 98%) |
| % Loop Modulated Basal ^c^ | - | 77% (66%, 89%) | - | 83% (73%, 88%) |

^a^ Values are median (Q1, Q3)

^b^ In the Medtronic only group, N=26 at baseline and N=29 at follow-up. In the Omnipod only group, N=337 at baseline and N=400 at follow-up.

^c^ Only those who completed 6 months follow-up included. N=30 for the Medtronic group and N=433 for the Omnipod group.
