# Supplementary material for: A Real-World Prospective Study of the Safety and Effectiveness of the Loop Open Source Automated Insulin Delivery System
Source: Diabetes Technol Ther. 2021 Apr 20;23(5):367–75. doi: 10.1089/dia.2020.0535 (PMC8080906; doi:10.1089/dia.2020.0535)
Supplement: Supplemental data [file Supp_Table7.docx]

# Supplemental Table S7. Insulin Metrics ^a^

|  | Overall | <7 Years | 7-<14 Years | 14-<25 Years | 25-<50 Years | ≥50 Years |
| --- | --- | --- | --- | --- | --- | --- |
| **24 Hours** |  |  |  |  |  |  |
| Total Daily Insulin (U/Kg) | 0.70 ± 0.40 | 0.79 ± 0.30 | 0.85 ± 0.24 | 0.81 ± 0.26 | 0.55 ± 0.53 | 0.47 ± 0.16 |
| Total Daily Basal Insulin (U/Kg) | 0.38 ± 0.26 | 0.37 ± 0.18 | 0.44 ± 0.15 | 0.43 ± 0.18 | 0.32 ± 0.36 | 0.26 ± 0.11 |
| Total Daily Bolus Insulin (U/Kg) | 0.33 ± 0.19 | 0.42 ± 0.20 | 0.41 ± 0.13 | 0.38 ± 0.15 | 0.23 ± 0.19 | 0.21 ± 0.10 |
| Basal/Bolus Ratio – Mean | 54%/46% | 46%/54% | 52%/48% | 53%/47% | 58%/42% | 56%/44% |
| **Daytime (6:00 AM – 11:59 PM)** |  |  |  |  |  |  |
| Total Daily Insulin (U/Kg) | 0.60 ± 0.34 | 0.70 ± 0.26 | 0.74 ± 0.21 | 0.69 ± 0.22 | 0.46 ± 0.44 | 0.39 ± 0.13 |
| Total Daily Basal Insulin (U/Kg) | 0.29 ± 0.20 | 0.29 ± 0.14 | 0.34 ± 0.12 | 0.32 ± 0.14 | 0.24 ± 0.27 | 0.19 ± 0.08 |
| Total Daily Bolus Insulin (U/Kg) | 0.31 ± 0.18 | 0.40 ± 0.19 | 0.40 ± 0.13 | 0.36 ± 0.15 | 0.22 ± 0.18 | 0.19 ± 0.09 |
| Basal/Bolus Ratio – Mean | 48%/52% | 41%/59% | 46%/54% | 47%/53% | 52%/48% | 51%/49% |
| **Nighttime (12:00 AM – 5:59 AM)** |  |  |  |  |  |  |
| Total Daily Insulin (U/Kg) | 0.13 ± 0.09 | 0.12 ± 0.09 | 0.14 ± 0.05 | 0.16 ± 0.05 | 0.12 ± 0.13 | 0.10 ± 0.05 |
| Total Daily Basal Insulin (U/Kg) | 0.09 ± 0.07 | 0.08 ± 0.05 | 0.11 ± 0.04 | 0.11 ± 0.04 | 0.09 ± 0.10 | 0.07 ± 0.03 |
| Total Daily Bolus Insulin (U/Kg) | 0.04 ± 0.03 | 0.04 ± 0.04 | 0.04 ± 0.02 | 0.05 ± 0.03 | 0.03 ± 0.03 | 0.03 ± 0.02 |
| Basal/Bolus Ratio – Mean | 71%/29% | 69%/31% | 75%/25% | 67%/33% | 72%/28% | 69%/31% |

^a^ Values are mean ± SD
