# Supplementary material for: A Real-World Prospective Study of the Safety and Effectiveness of the Loop Open Source Automated Insulin Delivery System
Source: Diabetes Technol Ther. 2021 Apr 20;23(5):367–75. doi: 10.1089/dia.2020.0535 (PMC8080906; doi:10.1089/dia.2020.0535)
Supplement: Supplemental data [file Supp_Table8.docx]

# Supplemental Table S8. System use among those using Loop at 6 months

|  | Months 1-6  N=481 |
| --- | --- |
| **CGM Use** |  |
| Median (IQR) | 96% (91%, 98%) |
| ≥90% | 365 (76%) |
| 80%-<90% | 55 (11%) |
| 70%-<80% | 29 (6%) |
| 60%-<70% | 16 (3%) |
| 50%-<60% | 5 (1%) |
| <50% | 11 (2%) |
| **Loop Modulated Basal** |  |
| Median (IQR) | 83% (73%, 88%) |
| ≥90% | 82 (17%) |
| 80%-<90% | 201 (42%) |
| 70%-<80% | 100 (21%) |
| 60%-<70% | 54 (11%) |
| 50%-<60% | 19 (4%) |
| <50% | 25 (5%) |
