# Supplementary material for: A Real-World Prospective Study of the Safety and Effectiveness of the Loop Open Source Automated Insulin Delivery System
Source: Diabetes Technol Ther. 2021 Apr 20;23(5):367–75. doi: 10.1089/dia.2020.0535 (PMC8080906; doi:10.1089/dia.2020.0535)
Supplement: Supplemental data [file Supp_Table9.docx]

# Supplemental Table S9. Device Discontinuations

|  |  |
| --- | --- |
| Number of participants who reported that Loop was discontinued | 15 (3%) |
| Reason for discontinuation ^a^ |  |
| I did not like it | 4 ( |
| I decided to try something else | 4 |
| It cost too much | 0 |
| It was hard to get all the supplies | 0 |
| Too complicated/couldn’t find resources to use it correctly | 4 |
| I didn’t see improvement in my blood sugar | 7 |
| It wasn’t working properly | 2 |
| My (or child’s) version was not up to date | 1 |
| I spent too much time on it | 5 |
| It just didn’t help as much as I thought | 9 |
| Had bad experience with user community | 0 |
| Concerned about disclosing use to doctor | 1 |
| Didn’t want to carry equipment around | 0 |
| No longer has diabetes | 0 |
| Other clinical trial participation | 1 |
| Pregnancy | 1 |
| Stopped until Omnipod available | 0 |

^a^ More than one reason could be reported for a discontinuation
