# Supplementary material for: A Real-World Prospective Study of the Safety and Effectiveness of the Loop Open Source Automated Insulin Delivery System
Source: Diabetes Technol Ther. 2021 Apr 20;23(5):367–75. doi: 10.1089/dia.2020.0535 (PMC8080906; doi:10.1089/dia.2020.0535)
Supplement: Supplemental data [file Supp_Table10.docx]

# Supplemental Table S10. Device Issues

|  | N=1587 |  |
| --- | --- | --- |
| **Device Type** |  |  |
| Apple Device | 27 (2%) |  |
| CGM | 382 (24%) |  |
| Infusion Set | 24 (2%) |  |
| Loop App | 269 (17%) |  |
| Pump | 142 (9%) |  |
| Rileylink | 241 (15%) |  |
| Undeterminable* | 499 (31%) |  |
| Other | 3 (<1%) |  |
| **Issue Type** |  |  |
| Algorithm, settings, incorrect data (excluding IOB) | 47 (3%) |  |
| Built in system error | 72 (5%) |  |
| Connectivity/Communication | 433 (27%) |  |
| Crashed, wouldn't open, froze OR Loading/processing/updating delay | 50 (3%) |  |
| Dexcom server availability | 25 (2%) |  |
| Error after software or firmware update | 34 (2%) |  |
| Failure to deliver requested bolus or delayed requested bolus | 87 (5%) |  |
| Hardware damage/failure | 154 (10%) |  |
| Inaccurate CGM Reading | 41 (3%) |  |
| Incorrect IOB value | 16 (1%) |  |
| Omnipod pairing | 63 (4%) |  |
| Settings erased | 13 (<1%) |  |
| Unknown problem resolved with restart/reset (and tune) RileyLink Only | 74 (5%) |  |
| Usability or user error | 59 (4%) |  |
| Other | 419 (26%) |  |
|  |  |  |

*Device issues for which attribution could not be made to one specific device
