# Supplementary material for: A Real-World Prospective Study of the Safety and Effectiveness of the Loop Open Source Automated Insulin Delivery System
Source: Diabetes Technol Ther. 2021 Apr 20;23(5):367–75. doi: 10.1089/dia.2020.0535 (PMC8080906; doi:10.1089/dia.2020.0535)
Supplement: Supplemental data [file Supp_Table11.docx]

# Supplemental Table S11. Safety Outcomes Through 6 Months Follow-Up

|  | Overall  N=558 | <7 Years  N=67 | 7-<14 Years  N=169 | 14-<25 Years  N=87 | 25-<50 Years  N=192 | ≥50 Years  N=43 |
| --- | --- | --- | --- | --- | --- | --- |
| **Severe Hypoglycemia** |  |  |  |  |  |  |
| Total # of events | 51 | 12 | 15 | 8 | 9 | 7 |
| # Events per participant |  |  |  |  |  |  |
| 0 | 523 (94%) | 59 (88%) | 159 (94%) | 83 (95%) | 184 (96%) | 38 (88%) |
| 1 | 22 (4%) | 6 (9%) | 5 (3%) | 1 (1%) | 7 (4%) | 3 (7%) |
| 2 | 11 (2%) | 1 (1%) | 5 (3%) | 2 (2%) | 1 (<1%) | 2 (5%) |
| ≥3 | 2 (<1%) | 1 (1%) | 0 (0%) | 1 (1%) | 0 (0%) | 0 (0%) |
| Incidence rate (per 100 person-years) | 18.7 | 36.6 | 18.3 | 18.6 | 9.6 | 32.8 |
| **Severe Hypoglycemia Resulting in a Seizure or Loss of Consciousness** |  |  |  |  |  |  |
| Total # of events | 5 | 0 | 1 | 0 | 3 | 1 |
| # Events per participant |  |  |  |  |  |  |
| 0 | 553 (>99%) | 67 (100%) | 168 (>99%) | 87 (100%) | 189 (98%) | 42 (98%) |
| 1 | 5 (<1%) | 0 (0%) | 1 (<1%) | 0 (0%) | 3 (2%) | 1 (2%) |
| 2 | 0 (0%) | 0 (0%) | 0 (0%) | 0 (0%) | 0 (0%) | 0 (0%) |
| ≥3 | 0 (0%) | 0 (0%) | 0 (0%) | 0 (0%) | 0 (0%) | 0 (0%) |
| Incidence rate (per 100 person-years) | 1.8 | 0.0 | 1.2 | 0.0 | 3.2 | 4.7 |
| **DKA** |  |  |  |  |  |  |
| Total # of events | 0 | 0 | 0 | 0 | 0 | 0 |
| # Events per participant |  |  |  |  |  |  |
| 0 | 558 (100%) | 67 (100%) | 169 (100%) | 87 (100%) | 192 (100%) | 43 (100%) |
| Incidence rate (per 100 person-years) | 0.0 | 0.0 | 0.0 | 0.0 | 0.0 | 0.0 |
| **Hospitalizations ^a^** |  |  |  |  |  |  |
| Total # of events | 20 | 1 | 3 | 3 | 12 | 1 |
| # Events per participant |  |  |  |  |  |  |
| 0 | 542 (97%) | 66 (99%) | 166 (98%) | 84 (97%) | 184 (96%) | 42 (98%) |
| 1 | 13 (2%) | 1 (1%) | 3 (2%) | 3 (3%) | 5 (3%) | 1 (2%) |
| 2 | 2 (<1%) | 0 (0%) | 0 (0%) | 0 (0%) | 2 (1%) | 0 (0%) |
| ≥3 | 1 (<1%) | 0 (0%) | 0 (0%) | 0 (0%) | 1 (<1%) | 0 (0%) |
| Incidence rate (per 100 person-years) | 7.3 | 3.0 | 3.7 | 7.0 | 12.7 | 4.7 |

^a^ Reasons for hospitalizations: pump site infection (1), ketonemia without DKA (3), nausea/vomiting not related to ketonemia (4), pregnancy deliveries (2), pregnancy-related (1), carpal tunnel surgery (2), colorectal surgery (1), cervical spine fusion (1), clotted dialysis graft (1), pneumonia (1), and unspecified reasons (3).
