# Supplementary material for: A Real-World Prospective Study of the Safety and Effectiveness of the Loop Open Source Automated Insulin Delivery System
Source: Diabetes Technol Ther. 2021 Apr 20;23(5):367–75. doi: 10.1089/dia.2020.0535 (PMC8080906; doi:10.1089/dia.2020.0535)
Supplement: Supplemental data [file Supp_Table12.docx]

# Supplemental Table S12. Cross Tabulation of Participants with ≥1 Severe Hypoglycemic Event Prior to and During the Study

|  | N ^a^ | Participants with ≥1 Event during 3 Months | Incidence Rate at 3 Months ^b^ | Participants with ≥1 Event during 6 Months | Incidence Rate at 6 Months ^b^ |
| --- | --- | --- | --- | --- | --- |
| Participants with ≥1 Event in 3 Months Prior to Enrollment | 97 | 16 (16%) | 91.1 | 20 (21%) | 64.8 |
| Participants with 0 Events 3 Months Prior to Enrollment | 448 | 12 (3%) | 13.5 | 14 (3%) | 8.6 |
| Total | 545 | 28 (5%) | 27.3 | 34 (6%) | 18.7 |

^a^ 18 participants who did not indicate the number of severe hypoglycemic events prior to enrollment on the baseline questionnaire are not included. One of them had a severe hypoglycemia event within 6 months.

^b^ Incidence reported per 100 person years
